# Carbapenem resistant *Campylobacter jejuni* bacteremia in a Bruton’s X-linked agammaglobulinemia patient

**DOI:** 10.1007/s10096-024-04937-1

**Published:** 2024-09-24

**Authors:** Ran Zhuo, Ramee L. Younes, Kevin Ward, Shangxin Yang

**Affiliations:** 1 Department of Pathology and Laboratory Medicine, UCLA David Geffen School of Medicine, University of California, 11633 San Vicente Blvd, Los Angeles, CA 90049 USA; 2 Division of Infectious Diseases, UCLA David Geffen School of Medicine, University of California, Los Angeles, CA USA

**Keywords:** Carbapenem resistance, *Campylobacter jejuni*, OXA-61, Gene duplication, Porin mutation, Recurrent bloodstream infections

## Abstract

Immunocompromised patients are prone to recurrent *Campylobacter* infections. We report a case of recurrent multi-drug resistant *Campylobactor jejuni* bloodstream infections in a Bruton’s X-linked agammaglobulinemia patient with prolonged ertapenem treatment. The isolate from the fifth recurrence developed carbapenem resistance, which is associated with mutations in a porin gene *porA*, and promoter changes and duplication of chromosomal bla*OXA-61* gene. Combination therapy using cefepime and doxycycline (later switched to moxifloxacin) cleared the infection.

## Introduction

Although campylobacteriosis is largely a self-limiting gastrointestinal illness in the immunocompetent, patients with hypogammaglobinemia are particularly prone to recurrent *Campylobacter bacteremia* that requires prolonged antibiotic treatment [1]. Resistance to first line treatment Ciprofloxacin and Erythromycin is increasing due to exposure to human antimicrobial use and practices in the poultry industry [2]. Carbapenems are often used in treating complicated extraintestinal *campylobacter* infections [3]. Multidrug resistant (MDR) *Campylobacter* species, more often *Campylobacter coli* strains, are emerging in Europe and Asia that are resistant to carbapenem, but the molecular mechanisms are not well defined [4–6].

We identified a MDR *Campylobacter jejuni* strain that developed carbapenem resistance in vivo after prolonged ertapenem treatment in a Bruton’s X-linked agammaglobulinemia patient and investigated the molecular mechanisms conferring antibiotic resistance. Genomic resistance information was used to guide treatment.

## Materials and methods

### Culture and phenotypic drug susceptibility tests

*Campylobacter jejuni* isolated from blood culture were identified by MALDI-TOF (BioMerieux VITEK MS v3.2). Susceptibilities were preliminarily obtained by direct disk diffusion method and concluded by broth microdilution testing which provides minimal inhibitory concentrations (MICs) endorsed by the Clinical and Laboratory Standards Institute (CLSI) guidelines.

### Whole-genome sequencing analysis

Isolate 1, 4 and 5 collected after the initial 1 week of ertapenem treatment (6/2022), 14 weeks (1/2024) and 16 weeks (3/2024) of cumulative ertapenem treatment were selected for whole genome analysis. Genomic DNA was extracted using Qiagen EZ1 tissue kit on the EZ1 Advanced XL instrument (Qiagen). DNA concentrations were measured using Qubit™ 1X dsDNA High Sensitivity Assay (ThermoFisher). Libraries were generated using the Revelo DNA-Seq Enz kit on MagicPrep™ NGS system (Tecan) and sequenced pair-ended on an Illumina MiSeq instrument (Illumina). Genome assembly and reads QC were performed using CLC Genomics workbench (Qiagen). Genomic resistance markers were predicted by using the build-in ResFinder and PointFinder databases in CLC Genomic workbench and confirmed using the Center for Genomic Epidemiology (CGE, https://www.genomicepidemiology.org/services/ accessed March 2024) and the Comprehensive Antibiotic Resistance Database (CARD, https://card.mcmaster.ca/ accessed March 2024). CGE was also used to determine the Multilocus sequence type (MLST) in silico. Mutational analysis and copy number calculation were performed using Geneious Primer software v2023.1.2 (Dotmatics). Gene copy number was estimated by the ratio of mean depth mapped to a specific gene vs. the entire reference genome.

## Results and discussion

**Fig. 1:**
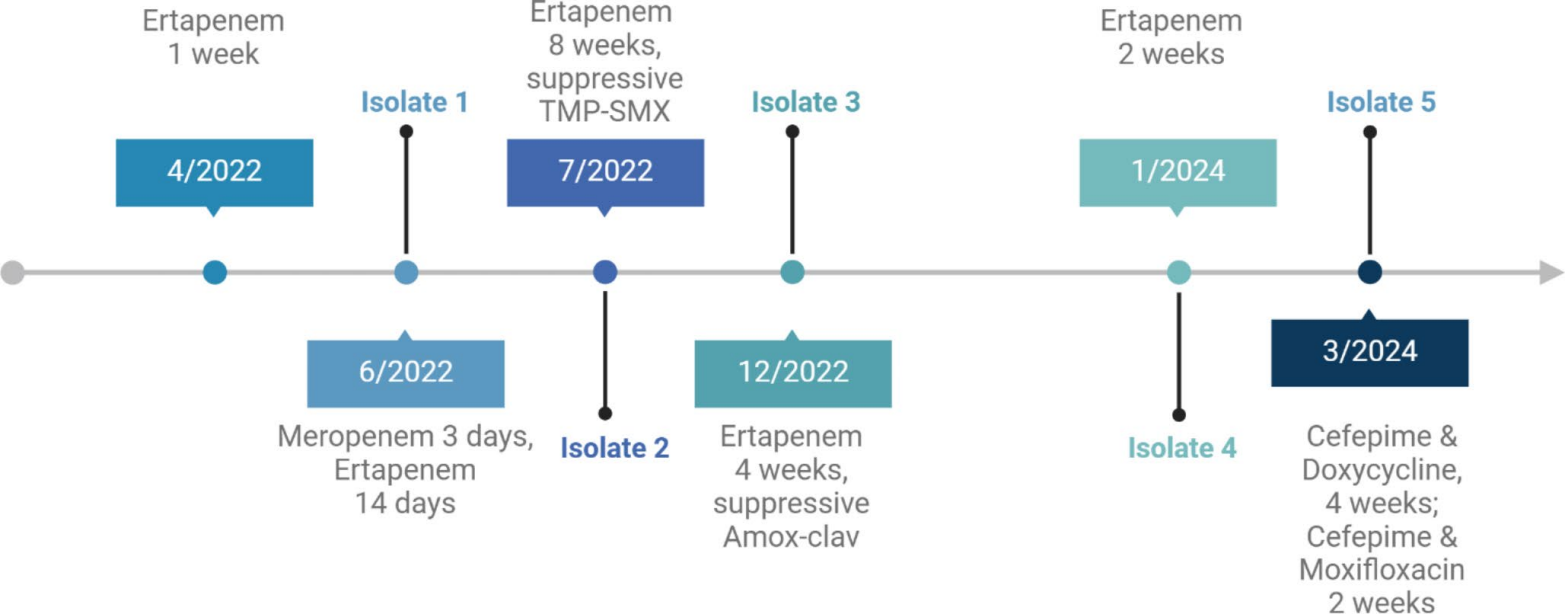
Recurrent *Campylobacter jejuni* bacteremia episodes and treatment timeline between April 2022 and March 2024

A 43-year-old man with Bruton’s X-linked agammaglobulinemia (XLA) complicated by XLA-related inflammatory bowel disease (on intravenous immunoglobulin every 3 weeks and chronic prednisone for several years), well-controlled HIV, and thrombocytopenia presented to our institution with a fifth recurrence of *Campylobacter jejuni* bacteremia. The patient first developed *Campylobacter*-associated diarrhea with concurrent bacteremia two years ago. He was treated at an outside hospital with ertapenem for 7 days due to ciprofloxacin resistance with some improvement. Three months later, his diarrhea and abdominal upset returned, with recurrence of *Campylobacter* bacteremia (Fig. 1, Isolate 1) for which he was referred to our hospital. He received two weeks of ertapenem, again with symptomatic improvement but bacteremia returned within 1 month (Fig. 1, Isolate 2). At that time, he received eight weeks of ertapenem therapy followed by planned, indefinite suppressive trimethoprim-sulfamethoxazole twice daily. Although he was initially responding well to this regimen, he was re-admitted 5 months later with a third recurrence of *Campylobacter* bacteremia (Fig. 1, Isolate 3), believed in part due to self-discontinuation of his suppressive antibiotic as it caused him abdominal discomfort. He then completed an additional 4-week course of ertapenem and transitioned to suppressive amoxicillin-clavulanic acid twice daily, which he tolerated well.

He was evaluated by Allergy/Immunology and was started on vedolizumab 7 months later as a steroid-sparing agent for management of his enteropathy with concurrent, gradual reduction in his chronic prednisone dosage. He experienced no further recurrence of bacteremia for 2 more months (Fig. 1, Isolate 4), when symptoms of diarrhea and abdominal pain developed despite compliance with suppressive amoxicillin-clavulanic acid. Ertapenem was once again selected, and the patient completed a 2-week course of ertapenem with initial resolution of symptoms but was admitted again due to recurrence of diarrhea while on ertapenem therapy. Blood cultures revealed an ertapenem-resistant *Campylobacter jejuni* isolate (Fig. 1, Isolate 5).

All five *C. jejuni* isolates were resistant to ciprofloxacin and azithromycin (Table 1). Isolate 5 has a 256-fold increase in ertapenem MIC and a 33-fold increase in meropenem MIC. The ertapenem susceptible Isolates 1 to 4 were resistant to tetracycline and gradually developed higher MIC to doxycycline. Intriguingly, the ertapenem resistant Isolate 5 became susceptible to both tetracycline and doxycycline.

**Table 1:** *Campylobacter jejuni* antimicrobial susceptibility of 5 isolates from blood culture

Drug	Antimicrobial susceptibility (MIC in mg/L), interpretation (if breakpoint available)				
	Isolate 1	Isolate 2	Isolate 3	Isolate 4	Isolate 5
**Collection Date**	6/15/2022	7/6/2022	12/9/2022	1/11/2024	3/6/2024
**Beta-lactam**					
Amoxicillin	4	4	8	-	> 8
Amox/clav	-	2	2	16	> 32
Ceftriaxone	8	8	8	2	8
Cefotaxime	2	4	2	0.5	> 8
Cefepime	-	-	-	≤ 0.5	≤ 0.5
Imipenem	-	-	-	≤ 1	≤ 1
Meropenem	≤ 0.06	≤ 0.06	≤ 0.06	≤ 0.06	2
Ertapenem	≤ 0.25	≤ 0.25	-	≤ 0.25	64
**Fluoroquinolone**					
Ciprofloxacin	4(R)	> 4(R)	> 4(R)	> 4(R)	4(R)
Levofloxacin	2	2	2	4	≤ 0.5
Moxifloxacin	-	1	1	1	1
**Tetracycline**					
Tetracycline	> 8(R)	> 8(R)	> 8(R)	> 8(R)	≤ 0.25(S)
Doxycycline	2(S)	2(S)	4(I)	4(I)	≤ 0.25(S)
**Macrolide**					
Erythromycin	16(I)	32(R)	-	32(R)	-
Azithromycin	> 32(R)	> 32(R)	-	> 32(R)	> 32(R)
**Aminoglycoside**					
Gentamicin	<=2	<=2	<=2	<=2	<=2
**Folic Acid Pathway**					
Trimethoprim-sulfamethoxazole	≤ 0.25/5	≤ 0.25/5	2/40	1/20	4/80

- Testing not performed

Interpretive criteria are defined by the CLSI breakpoints

Isolate 1, 4 and 5 selected for whole genome analysis are of the same multi-locus sequence type ST-22, with less than 22 SNPs difference, suggesting they are the same strain that evolved under antibiotic selective pressure (Table 2). All three isolates carry a T86I mutation in gyrase A, conferring resistance to fluoroquinolones, and a A2075G mutation in the 23 S rRNA gene, conferring resistance to macrolides; however, erm(B) gene was not found in any of the isolate [2, 7]. Isolate 1, 4 and 5 also have two point-mutations, T-35 C and T-40 A, in the CmeR binding site upstream of the *cme ABC* operon, which encodes the resistance-nodulation-cell division (RND) efflux system in *Campylobacter* [8, 9]. The T-35 C mutation abolishes the binding of CmeR to the inverted repeat (IR) and leads to overproduction of CmeABC [9]. The T-40 A mutation is a previously unknown point mutation in the same IR that may result in further inactivation of CmeR binding. CmeABC multidrug efflux pump is known to contribute to the resistance to beta-lactams, tetracyclines, fluoroquinolones, and macrolides [5, 10].

**Table 2:** Genetic determinant of antimicrobial resistance in *Campylobacter jejuni* isolate 1,4 and 5

Genetic component	Iso 1	Iso 4	Iso 5	Confer resistance to
MLST serotype	22	22	22	
SNP distance from Isolate 1	0	20	22	
**OXA-61 family D beta-lactamase**				Beta-lactams
blaOXA-61 promoter TATA box (G-57T)	No	Yes	Yes	
blaOXA-61 promoter RBS (A-9G)	No	Yes	Yes	
blaOXA-61 family D beta-lactamase (G715A/Glu239Lys)	No	Yes	No	
blaOXA-61 family D beta-lactamase (G715A&A716G/Glu239Arg)	No	No	Yes*	
Copies of OXA-61	1	1	3*	
**Major Outer Membrane protein (MOMP)**				Beta-lactams, Tetracyclines, Macrolides
porA (Ile135Thr)	No	No	Yes*	
porA (Gly141Asp)	No	No	Yes*	
porA (Asp302Gly)	No	Yes	No	
**Efflux pump cmeABC repressor cmeR**				Beta-lactams, Fluoroquinolones, Tetracyclines, Macrolides
cmeR binding site palindromic sequence (T-40 A& T-35 C)	Yes	Yes	Yes	
**Tetracycline**				
tet(O)	Yes	Yes	No*	Tetracyclines
**Fluoroquinolone**				
gyrA (gyrA p.T86I, ACA-> ATC)	Yes	Yes	Yes	Fluoroquinolones
**Macrolide**				
23 S rRNA (23 S r.A2075G)	Yes	Yes	Yes	Macrolides

*Unique genotype to Isolate 5 which is carbapenem resistant

Tetracycline resistance is common in *Campylobacter* and is conferred by the tet(O) gene by modifying the target ribosomal A site via TetO binding [11, 12]. Interestingly, tet(O), a plasmid-borne gene [13], was present in the tetracycline resistant Isolate 1 and 4 but was lost in Isolate 5 which became tetracycline susceptible.

Two nonsynonymous mutations both at amino acid (aa) position 239 of the OXA-61 were identified in Isolate 4 (G715A/Glu239Lys) and Isolate 5 (G715A&A716G/Glu239Arg). The Glu239Lys mutation is found in another MDR *Campylobacter jejuni* strain from a similar published case [4]; the Glu239Arg is a novel mutation at the same amino acid position. Both mutations swapped a positively charged amino acid to a negatively charged amino acid. Their role in carbapenem resistance is undefined.

G to T transversion that restores the TATA box at -57 and a A to G transversion at -9 promoter region of the OXA-61 like gene were also found in Isolate 4 and 5 but not Isolate 1. These mutations were associated with high-level beta-lactam resistance [14]. Two extra copies of blaOXA-61 were present exclusively in isolate 5 (Table 2). In addition, two nonsynonymous mutations in PorA protein were identified in Isolate 5. Nunes et al. reported an in-frame Asp insertion at aa 139 which contributed to resistance to ertapenem and meropenem [4]. The mutations in Isolate 5 are found at position 135 and 141, both substituted a nonpolar residual to polar residuals. Mutations in *porA* may decrease the permeability of carbapenem on the bases of charge or size [5, 15, 16].

To ensure clearance, a combination therapy regimen was selected. Although doxycycline is highly bioavailable, it does not reach effective concentrations in the serum to be used reliably as monotherapy. Given the resistance to ciprofloxacin and gyrase mutation, there was concern for rapid development of further quinolone resistance with single-agent moxifloxacin or levofloxacin. Due to the high MIC to ertapenem and porin mutation, there was also concern for class-wide carbapenem resistance. The patient was given cefepime and doxycycline for a planned 4-week course, which the patient completed with near complete resolution of symptoms, although he noted bothersome photosensitivity while on doxycycline. He was then transitioned to cefepime and moxifloxacin combination therapy for an additional 2 weeks with complete resolution of symptoms. During this treatment period, his oral prednisone dosage was further tapered to 1 mg daily.

Several studies have shown the efficacies of regimens for multidrug resistant (MDR) *Campylobacter* including carbapenems, aminoglycosides, advanced tetracycline derivatives, and beta-lactam/inhibitor combinations such as amoxicillin-clavulanic acid [15, 17–19]. However, breakpoints are only available for macrolides, ciprofloxacin, and tetracyclines per CLSI, with the addition of meropenem breakpoints in the next edition of M45. In this report, after prolonged ertapenem treatments (18 weeks in total), the patient developed a new episode of bacteremia caused by a carbapenem-resistant *C. jejuni*. Similar cases of carbapenem-resistant *Campylobacter coli* causing recurrent bacteremia in immunocompromised patients have been reported before [4, 5].

Genomic analysis revealed that the derepression of CmeABC efflux pump operon, overexpression of OXA-61 by restoring a cryptic TATA box and increased blaOXA-61 gene copy number, and genetic alterations in the major outer membrane protein porA may collectively lead to carbapenem resistance seen in Isolate 5. However, these genetic changes did not alter the susceptibility to cefepime, as evidenced by the consistent low MIC and the successful treatment using cefepime as the backbone drug in this case.

Carbapenems are the drug of choice for severe infections caused by MDR *Campylobacter*, however, carbapenem resistance in *Campylobacter* is emerging. This case highlights the need for surveillance of susceptibility to carbapenem and reporting in *Campylobacter*. Eradication of intestinal carriage of *Campylobacter* with intravenous and oral antibiotic therapy may help control recurrent infections in immunocompromised patients and the selection of drugs should be guided by phenotypic susceptibility testing and genomic resistance profile analysis using whole genome sequencing when available.

## Data Availability

Data is available upon request.
